# Systematics of *Simplicia* Kirk (Poaceae, Agrostidinae) – an endemic, threatened New Zealand grass genus

**DOI:** 10.3897/phytokeys.75.10328

**Published:** 2016-12-09

**Authors:** Peter J. de Lange, Rob D. Smissen, Jeremy R. Rolfe, Colin C. Ogle

**Affiliations:** 1 Terrestrial Ecosystems, Science and Policy Group, Department of Conservation, Private Bag 68908. Auckland 1145, New Zealand; 2 Allan Herbarium, Landcare Research, P.O. Box 69, Lincoln 7640, New Zealand; 3 Terrestrial Ecosystems, Science and Policy Group, National Office, Department of Conservation, P.O. Box 10420, Wellington 6143, New Zealand; 4 22 Forres Street, Whanganui, New Zealand

**Keywords:** Poaceae, Simplicia, new species, Simplicia felix, Simplicia buchananii, Simplicia laxa, conservation status, New Zealand flora

## Abstract

A new species of the New Zealand endemic grass *Simplicia*, *Simplicia felix* is described. The new species is segregated from and compared with *Simplicia buchananii* and *Simplicia laxa*. *Simplicia felix* occurs mostly in lightly shaded areas of seasonally dry alluvial forest. A distribution map and an assessment of the conservation status of the new species are presented. Genetic variation in the genus was examined, building on previously published work but including additional sampling. Analysis of nrDNA ITS and ETS and plastid *trn*L intron and *trn*L–F intergenic spacer sequences show *Simplicia felix* to be more closely related to *Simplicia laxa* than to *Simplicia buchananii*. NeighborNet analyses of AFLP profiles for the three species of *Simplicia* show each to consist of distinct clusters of genotypes well separated from each other.

## Introduction

**Figure 1.**
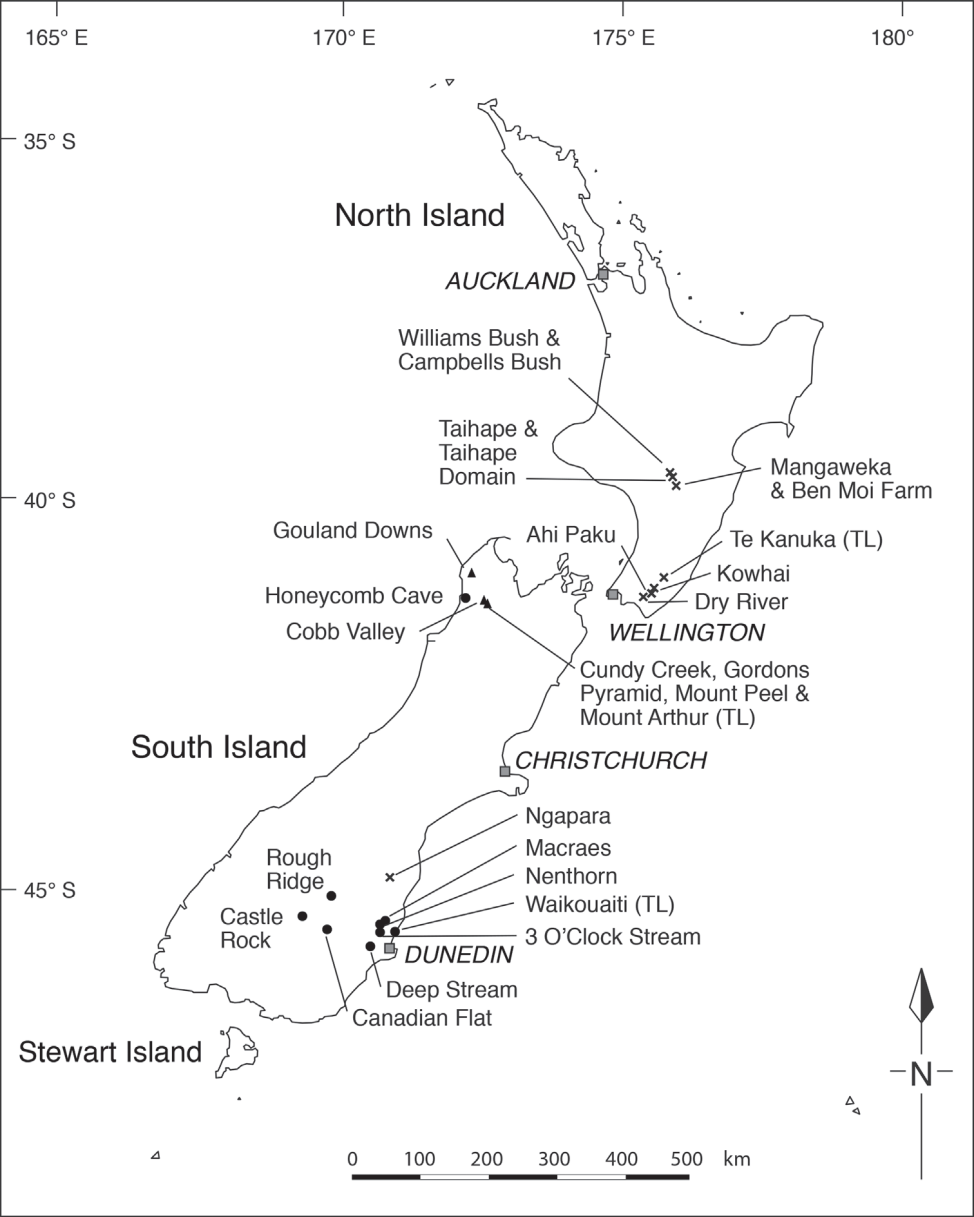
Distribution of *Simplicia* species and showing locations discussed in text.

During 2005 one of us (Colin Ogle), chanced upon an unremarkable grass growing in an alluvial forest remnant on the margin of a grazed pasture in the Kawhatau Valley Mangaweka, in the Rangitikei District, North Island (Ogle 2010) (Fig. 1). When the identity of the grass could not be satisfactorily determined, the specimen was sent to various colleagues whereupon it was eventually determined as a species of the endemic ditypic New Zealand genus *Simplicia* Kirk. There the matter would have rested if it were not for the fact that there was no consensus on the identity of the species. The *Simplicia* had features in common with both *Simplicia buchananii* (Zotov) Zotov and *Simplicia laxa* Kirk.

To resolve the matter a DNA-based investigation of the genus was initiated to try to determine which species the plant actually was (Smissen et al. 2011). That study, utilising nuclear ribosomal DNA (nrDNA) sequences, plastid DNA sequences, and AFLP profiles, found that, while the North Island *Simplicia* was ‘referable to *Simplicia laxa*’, those plants were genetically distinct from other South Island *Simplicia laxa* populations (Smissen et al. 2011). A complication was that nrDNA sequence for a *Simplicia* sample from Ngapara (North Otago) that was not included in the AFLP study suggested it was more closely related to the North Island *Simplicia* than to other South Island samples (Fig. 1), Although Smissen et al. (2011) suggested a taxonomic revision of the genus was warranted, they advised that AFLP data needed to be obtained from the North Otago (Ngapara) population to confirm its relationship with North Island samples indicated by nrDNA sequences. They also recommended that a survey for *Simplicia* within the Wairarapa (Fig. 1) of the North Island be carried out. It was in the eastern Wairarapa during 1880, that Thomas Kirk first discovered the genus (Kirk 1897) and, whilst Kirk referred his Wairarapa material to *Simplicia laxa* (which he described from plants collected in 1880 by Donald Petrie from Waikouaiti (Fig. 1) on the north-eastern Otago coastline, South Island), his herbarium material seemed to be closer to Ogle’s potentially ‘new’ *Simplicia* than to *Simplicia laxa* sens. str. When Smissen et al. (2011) concluded their study, 131 years had elapsed since *Simplicia* had been seen in Wairarapa, and Kirk’s herbarium material was unlikely to yield suitable DNA for sequencing or AFLP profiling.

During February 2014 *Simplicia* was rediscovered in the Wairarapa (de Lange et al. 2014), from which fresh material and herbarium specimens were obtained. These enabled us to generate new genetic data for these plants and also a plant we received from Ngapara, for which we could now generate AFLP profiles and a full set of DNA sequences. This additional data confirmed that the Wairarapa and Ngapara *Simplicia* were part of the same genetic cluster as those found in the Mangaweka–Taihape area.

That data, along with morphological evidence, suggests that the recognition of a third species of *Simplicia* and a narrower circumscription of *Simplicia laxa* are justified.

## Materials and methods

### Molecular phylogenetics

Details of samples and GenBank accessions are shown in Table 1.

**Table 1.** Sample details.

Species	Location	Region	Lat/long	vouchers	GenBank ITS	GenBank ETS	GenBank *trn*L/F
*Simplicia buchananii*	Cobb Valley	Nelson	41°7'50.96"S, 172°36'31.53"E	No Voucher	HM191441	HM191453	HM191463
*Simplicia buchananii*	West of Gordon’s Pyramid	Nelson	41°11'15.24"S, 172°40'3.32"E	AK 304801	HM191439	HM191451	HM191465
*Simplicia buchananii*	Cundy Creek	Nelson	41°11'14.32"S, 172°40'02.28"E	AK 304802	HM191440	HM191452	HM191464
*Simplicia felix*	Williams’s Bush	Wellington	39°37’ 20.7"S, 175°45'54.02” E	CHR 607323	HM191437	HM191449	HM191461
*Simplicia felix*	Taihape Reserve	Wellington	39°40'14.29"S, 175°48'18.96"E	CHR 607322	HM191435	HM191447	HM191459
*Simplicia felix*	Ben Moi Farm	Wellington	39°46'50.25"S, 175°51'59.14"E	CHR 607321	HM191438	HM191450	HM191462
*Simplicia felix*	Kaumingi 1	Wellington	40°57’ 50.18"S, 175°52’ 15.83"E	AK 351330	KU724189	KU728132	KU728136
*Simplicia felix*	Kaumingi 2	Wellington	40°58’ 4.56"S, 175°52’ 26.49"E	AK 351325	KU724190	KU728131	KU728135
*Simplicia felix*	Te Kowhai	Wellington	41°10’ 57.62"S, 175°40’ 59.85"E	AK 351290	KU724191	KU728130	KU728134
*Simplicia laxa*	Honeycomb Cave	Nelson	41°8'25.79"S, 172°11'42.41"E	AK 288071 CHR 607318	HM191442	HM191454	HM191466
*Simplicia felix*	Ngapara	Otago		AK285424	KU724188	KU728129	KU728133
*Simplicia laxa*	Nenthorn	Otago	45°28'39.6"S, 170°22'47.3"E	CHR 607324 CHR 607326 CHR 607327 CHR 607328 CHR 607329	HM191445	HM191457	HM191470
*Simplicia laxa*	Macraes	Otago	45°25'35.95"S, 170°27'13.9"E	CHR 607319 CHR 607320	-	-	-
*Simplicia laxa*	Castle Rock	Otago	45°17'53.92"S, 169°16'13.87E	AK 304847 AK 304848	HM191443 HM191446	HM191455 HM191458	HM191467 HM191469

For DNA sequencing methods see Smissen et al. 2011. New sequences for the *trn*L intron, *trn*L–*trn*F intergenic spacer, nuclear ribosomal (nrDNA) ITS and nrDNA ETS regions were generated for three Wairarapa *Simplicia* samples and one from Ngapara (Otago) and aligned with those from our previous study (see table 1). Outgroup sequences were those used in Smissen et al. (2011). Gaps were coded as binary characters using the method of Simmons and Ochoterena (2000) as implemented in Fastgap1.2 (Borchsenius 2009). Congruence between DNA regions was not re-assessed since new sequences were closely similar to those included in Smissen et al. (2010). DNA sequences were analysed under parsimony using PAUP 4.0b10 (Swofford 2003). A heuristic search strategy using default settings was employed. Trees were condensed to collapse branches where minimum branch length was 0. Bootstrap values were calculated from 1000 replicates saving a maximum of 10000 trees for each replicate.

New AFLP profiles were generated from the same three Wairarapa samples plus an additional sample (see Table 1) along with new AFLP profiles for a representative group of existing DNA samples from our previous study. One DNA sample (Ngapara) was subjected to duplicate DNA extraction and digestion/ligation, another (Te Kowhai) to duplicate digestion/ligation. All samples were duplicated for PCR amplification (preamp and selective), capillary separation and scoring. AFLP protocols followed the same method as Smissen et al. (2011) except that selective primers targeting EcoRI restriction sites were labelled with 6-FAM or VIC fluorescent dyes, and fragments were separated by capillary electrophoresis on an ABI3500XL genetic analyser using ROX labelled GS500 size standard instead of polyacrylamide slab gels. The selective primer combinations used were Eco-ACG with Mse-CAG and Eco-AAC with Mse-CTC. Automated scoring was conducted using GeneMapper 4.1 (ABI) starting at 100 bp and finishing at 500bp, using a bin width of 0.5 (see Holland et al. 2008), peak threshold of 30, signal strength among samples normalised using the sum of signalsetting, and max peak width of 1.5. We called alleles in three states based on the maximum peak intensity for each bin such that samples displaying peaks < 10% of the max intensity were scored as absent (0), samples displaying peaks > 10% but < 20% were scored as ambiguous (?) and samples with peaks > 20% were scored as present (1). AFLP profiles were analysed by NeighborNet as implemented in Splitstree 4.10 (Huson and Bryant 2006) using p-distances.

### Plant collections and specimens

Descriptions of *Simplicia buchananii*, *Simplicia laxa* sens. str. and the new species were prepared using cultivated plants and herbarium specimens held at AK, CHR and WELT (herbarium acronyms follow Thiers (2016). Live plants of *Simplicia laxa* sens. str. and the new species were cultivated for this study at Oratia Native Plant Nursery, West Coast Road, Oratia, West Auckland. Measurements of spikelets, florets, fruits and hairs were obtained using a binocular Leica Wild M3C light microscope fitted with a graticule, which had been calibrated by staff at AK. Leaf, culm and inflorescence measurements were obtained using digital calipers (Mitutoyo Digimatic 500-321 CD–6). Measurements are based on a full sampling of the Herbarium specimens available, usually with 5–10 measurements made per specimen. The sole exception are those measurements obtained for the caryopsis of the three *Simplicia*, for which only limited examples were available (*Simplicia buchananii* *n* = 2, *Simplicia laxa* sens. str. *n* = 4, *Simplicia* sp. nov. *n* = 5)

## Results

### DNA sequences

Little resolution was obtained by phylogenetic analysis of the plastid DNA sequences which included only 12 variable characters (four with outgroups excluded), only five of which were parsimony informative (three with outgroups excluded). In the single most parsimonious tree (length 13) found by heuristic search of these (not shown) the three *Simplicia buchananii* samples are characterised by two synapomorphic substitutions in the region sequenced. Some but not all of the North Island *Simplicia laxa* samples share a single synapomorphic substitution.

**Figure 2.**
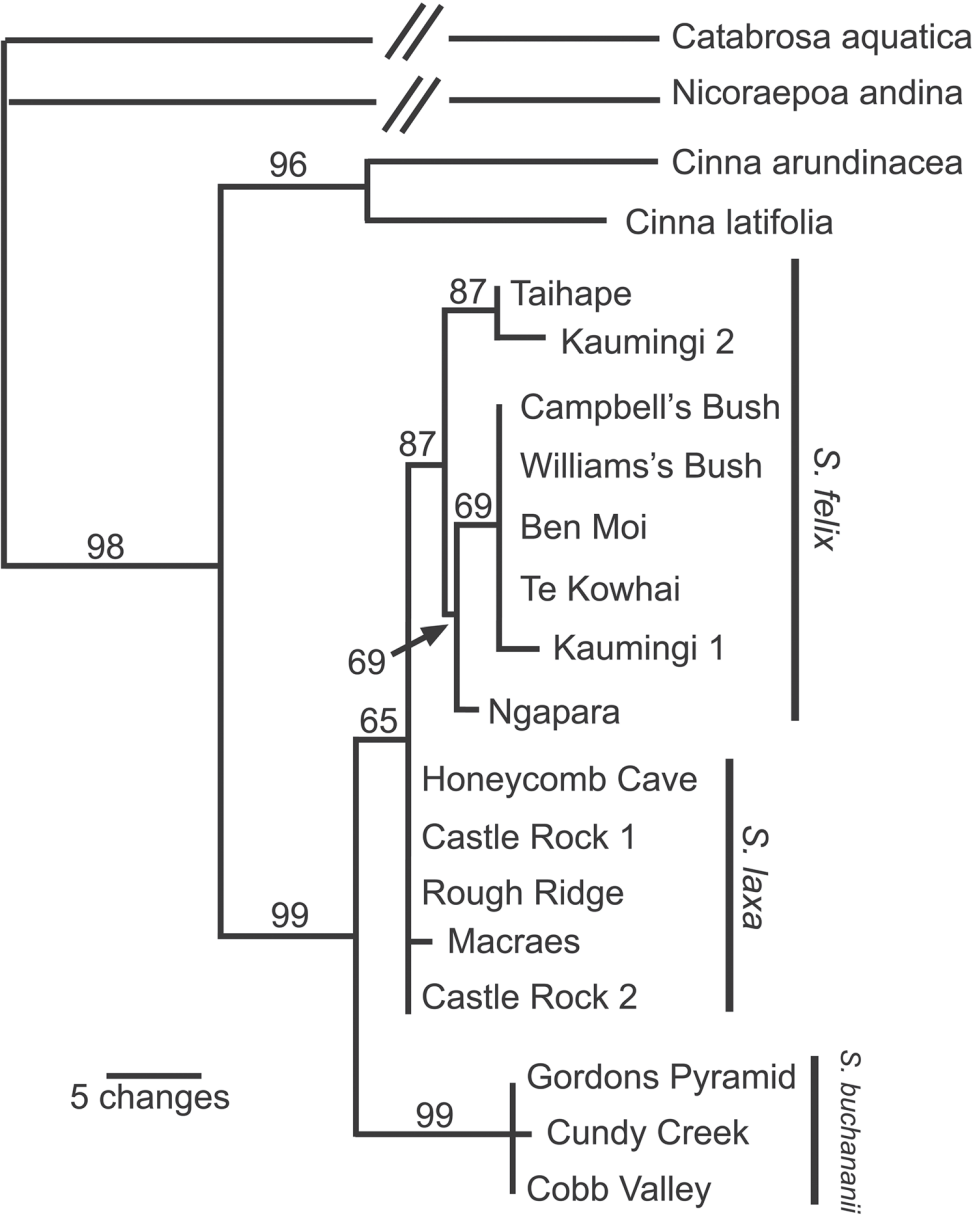
Selected most parsimonious tree for nrDNA sequence data. Numbers above branches are bootstrap percentages. Diagonal lines indicate that the branches leading to the outgroup *Nicoraepoa* and *Catabrosia* sequences are not shown to scale.

More resolution was obtained in analysis of the nrDNA sequences which contained 196 variable characters (25 with outgroups excluded), 53 of which were parsimony informative (20 with outgroups excluded). Three equally parsimonious trees (length 218) were recovered by the heuristic search. One of these (Fig. 2) has the same topology as the strict consensus of most parsimonious trees. In all the most parsimonious nrDNA trees *Simplicia buchananii* and *Simplicia laxa* *sensu lato* are recovered as reciprocally monophyletic sister groups which received 99% and 95% bootstrap support respectively. Within *Simplicia laxa* *sensu lato* all the North Island samples together with the Ngapara sample form a clade excluding the other South Island samples (bootstrap support 87%). For ease of readability the new name *Simplicia felix* is used for the North Island/Ngapara clade in the following Results and Discussion, and the name *Simplicia laxa* is used for the South Island clade.

### Accounting for scoring error in AFLP

**Figure 3.**
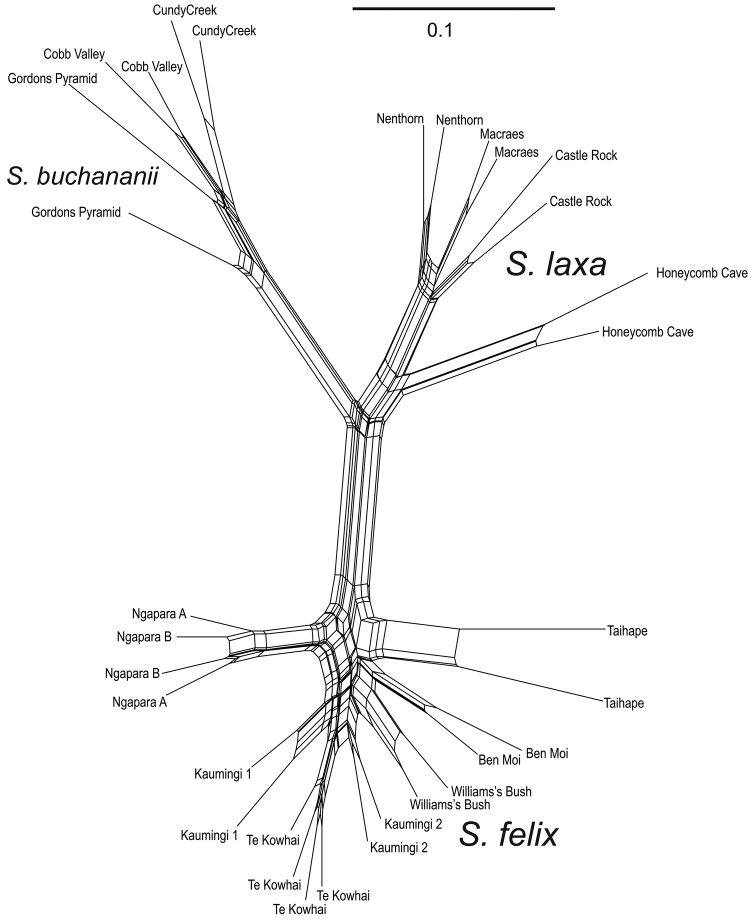
NeighborNet graph for AFLP data with all automatically scored polymorphisms.

**Figure 4.**
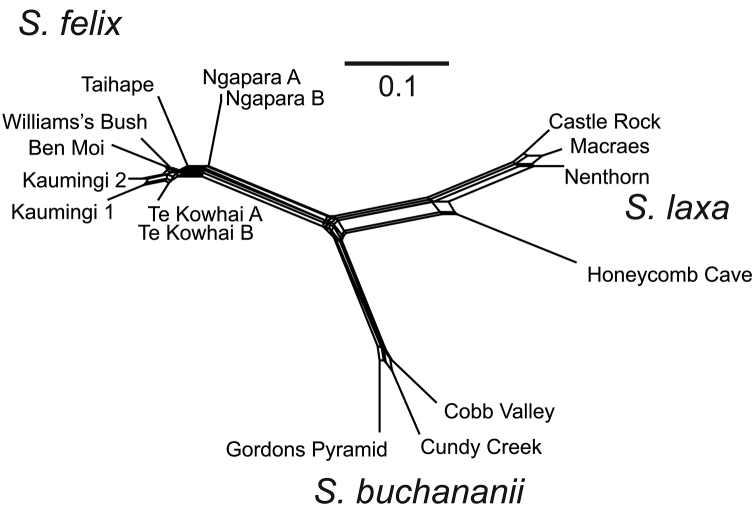
NeighborNet graph for reduced AFLP data with reproduced polymorphisms only.

Fragment scoring with the settings described above resulted in data matrices with 475 fragments for Eco-AAC with M-CTC and 559 for Eco-ACG with Mse-CAG. NeighborNet graphs generated for each primer pair showed similar grouping of samples (not shown) and we combined them to form a single data matrix with 1034 fragments. The NeighborNet for the full data matrix is shown in Fig. 3. Samples are grouped into three major groups; all North Island *Simplicia laxa* samples together with the Ngapara sample, the Nelson *Simplicia buchananii* samples, and the South Island *Simplicia laxa* samples (i.e., excluding the Ngapara sample). It is apparent that replicate amplifications of the same DNA sample did not return the same scored genotype. Pairwise differences between replicate amplifications of the same sample ranged from 6.1% to 17.2%. Examination of the chromatograms suggests that at least most of the difference between replicates was due to differences in automated scoring as a result of binning and threshold effects rather than problems with the underlying chemistry producing different sets of fragments. The samples replicated from the DNA extraction or digestion/ligation stages were not notably more different than the remainder which were only duplicated from preamp stage on. We have eliminated this scoring error through a two-step process. Firstly, we deleted from the data matrix all those bins where any sample displayed a peak with intensity ranging from 10 to 20% of the highest peak scored in that bin from any sample. We then deleted any remaining bins that were scored as present in one replicate and absent in the other replicate for any sample. This left a much reduced matrix of 204 characters that were consistently scored between duplicates. A NeighborNet graph generated for this data set (Fig. 4) recovered the same major groups as the analysis with all automatically scored fragments.

### AFLP groupings

Three major groups are obtained in both NeighborNet analyses of AFLP profiles. One group corresponds with *Simplicia buchananii* including samples from Gordons Pyramid, Cobb Valley, and Cundy Creek (see Fig. 4). Samples of *Simplicia laxa* sens. lat. are split into two groups. The first group, now *Simplicia felix*, includes all the North Island samples and the sample from Ngapara. The second group, *Simplicia laxa*, includes the Honeycomb Cave sample and the remaining Otago samples.

## Discussion

Relative to eyeball scoring of silver-stained slab gels, fluorescent labelling and automated scoring AFLP profiles produces many more apparent polymorphisms but with considerably reduced profile reproducibility, at least for *Simplicia* in our hands (see Smissen et al. 2011). At face value, the error rates reported here (6.1% to 17.2%) are high compared to those reported by other studies (see Meudt and Clarke 2007) and could perhaps be lowered by adjusting scoring parameters. Instead, since most of this reduction in reproducibility appears to be the result of threshold and binning effects in automated scoring, much of it can be accounted for by duplicating samples and using a scoring protocol recording peaks of low–intermediate intensity as uncertain. Analyses of our AFLP profiles using all scored polymorphisms or just reproducible polymorphisms yield essentially similar results in terms of relationships among plants, but the former approach exaggerates the level of variation within genetic clusters of individuals at the expense of the level of variation among these clusters.

The results reported here are in accord with those obtained by Smissen et al. (2011) that suggested two distinct genetic groups are included within the current circumscription of *Simplicia laxa*. Additionally, we show here that the sampled Wairarapa plants of *Simplicia* are most closely related to Taihape area and Ngapara (Otago) plants (*Simplicia felix* sp. nov.), and less closely related to those from Honeycomb Cave (Nelson) and the remaining plants sampled from Otago (*Simplicia laxa* sens. str.). The presence of these two genetic groups, distinguishable by AFLP profiles and nrDNA sequences, suggests that more than one taxon might be recognised within the current circumscription of *Simplicia laxa*.

Seed set in isolated, glasshouse-grown plants indicate that both *Simplicia buchananii* and *Simplicia laxa* are self-compatible (Zotov 1971; Connor 1988). Moreover, Connor (1988) stated that their flower morphology suggests that self-fertilization is “not-infrequent”. It is possible that outcrossing is naturally rare in *Simplicia* and that genetic races could therefore persist in sympatry through a preponderance of selfing. The relatively low levels of genetic distance within the genetic groups of *Simplicia laxa* compared to the distance between them (Fig. 4) is consistent with a predominantly uniparental reproductive strategy resulting in strong genetic bottlenecks. Therefore, the coexistence in Otago of two genotypes of *Simplicia laxa* sens. lat. does not necessarily imply they should be recognised at species, or indeed any rank. On the other hand, the genetic (and morphological) differences between the two groups appear to be substantial, compared to the variation within each and there are consistent, if subtle, morphological differences between them. Therefore we recognise them here at species rank. Zotov’s (1971) lectotypification of *Simplicia laxa* using Petrie’s Waikouaiti plants means that those plants with hairy culms, leaves and inflorescences, and larger inflorescences encompass *Simplicia laxa* and that the remaining plants require a new name (*Simplicia felix*). The necessary action to formalize this name is taken below.

## Systematics

### Simplicia

Taxon classificationPlantaePoalesPoaceae

Kirk, T.P.N.Z.I. 29: 497 (1897)

#### Type.

*Simplicia laxa* Kirk (fide Zotov 1971)

#### Key to New Zealand *Simplicia*

**Table:** 

1	Plants tufted; culms erect, up to 1 m tall (culm nodes not root-forming); inflorescences erect, linear, branches, erect, appressed to rachis, bearing spikelets almost to base	***Simplicia buchananii***
–	Plants decumbent; culms weakly ascendant, rooting freely from culm-nodes so forming diffuse interconnected widely sprawling clonal patches 0.6–1.0 m diameter; inflorescences linear to pyramidal, binate, basal branch or branches reflexed, devoid of spikelets from lower ½ to ⅔	**3**
2	Mid-stem and upper stem leaf sheaths finely ribbed, copiously hairy (hairs 0.35–0.40 mm long); adaxial leaf-blade ribs hairy; inflorescence branches antrorsely hairy, pedicels 1.00–1.06 mm long; lemma pubescent	***Simplicia laxa***
–	Mid-stem and upper stem leaf sheaths strongly ribbed, ± glabrous (occasionally bearing minute hairs towards sheath apex); adaxial leaf-blade ribs smooth or finely scabrid; inflorescence branches scabrid, pedicels 0.20–0.30 mm long; lemma minutely scabrid	***Simplicia felix***

### The species

#### Simplicia buchananii

Taxon classificationPlantaePoalesPoaceae

(Zotov) Zotov, New Zealand J.Bot. 9: 542 (1971)

≡ Poa uniflora Buchanan Indig. Grasses N.Z. t49B (1880) non Muhl. (1817)
≡ Simplicia laxa var. buchananii Zotov T.R.S.N.Z. 73: 236 (1943)

##### Holotype.

‘Mt Arthur’ *A. McKay s.n.*, 1874, (WELT SP059605!) (*fide* Zotov 1971).

##### Etymology.

Named by Zotov (1971) for John Buchanan FLS (1819–1898), Scottish born draughtsman, and New Zealand’s first government employed botanist (Taylor 2002). Buchanan first described *Simplicia buchananii* as a species of *Poa*, *Poa uniflora* Buchanan but that species name was preoccupied (*Poa uniflora* Muhl.) (Zotov 1971).

##### Description

**Figure 5.**
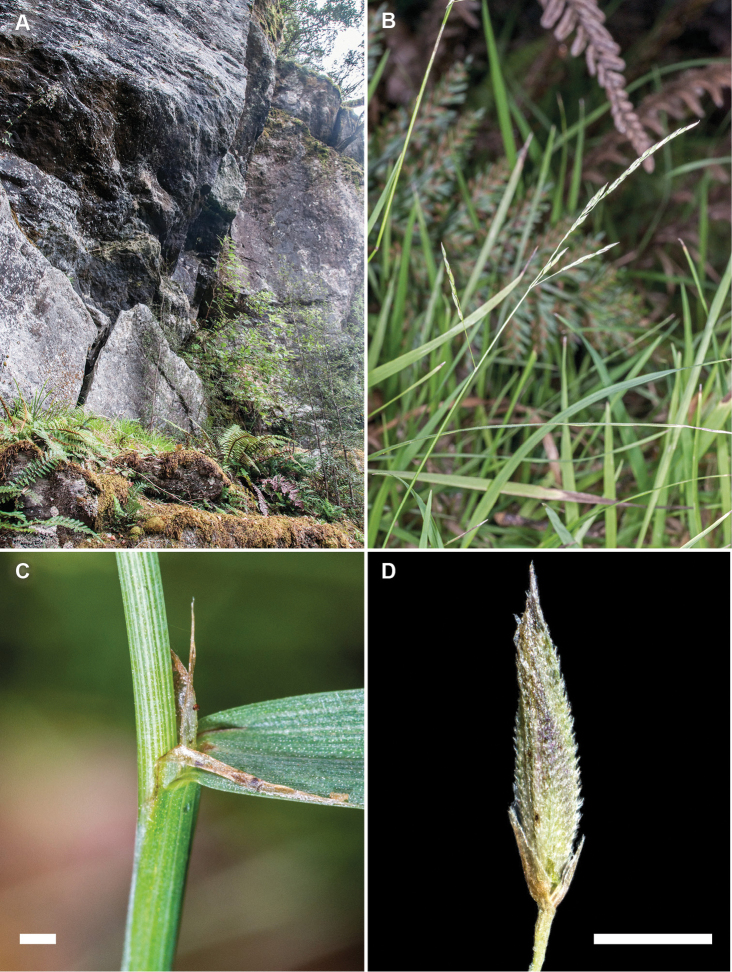
*Simplicia buchananii*. **A** habitat, Kahurangi National Park, North West Nelson, South Island, New Zealand **B** growth habit and inflorescence **C** culm, leaf base, sheath and ligule **D** spikelet showing reduced glumes and lemma (images: J.R. Rolfe).

**(Fig. 5).** Plants gracile, tufted, 0.40–0.60(–1.0) m tall. Culms 0.40–0.80 m long, bright green when fresh, wiry, erect (sometimes with apices weakly pendant), culm internodes 3–5, elongated, glabrous; internodes ± equal in length to subtending leaf-sheaths. Culm-nodes slightly swollen when fresh, glossy orange-brown to dark red-brown (0.1–)0.3–0.4(–0.5) mm long. Basal leaf-sheaths stramineous or dull brown, membranous, strongly ribbed, usually glabrous, sometimes scabrid on ribs or evenly, finely pubescent; hairs when present retrorse to patent, minute (0.06–0.08 mm long); mid stem and upper leaf-sheaths stramineous to green, membranous, strongly ribbed, glabrous. Ligule 2.0–3.5(–4.0) mm, membranous, lanceolate, apex erose to very deeply lacerate; glabrous. Leaf-blade 100–200 × (1.8–)3.0(–4.0) mm, green to yellow-green, flat, linear-lanceolate with acuminate apices, finely ribbed; adaxial ribs finely scabrid, abaxial ribs glabrous; margins smooth. Panicle (40–)160(–180) mm long, linear; rachis glabrous, branches (10–)30(–36) mm long, erect, glabrous (sometimes bearing minute antrorse prickle-teeth near pedicels), bearing spikelets almost to base, pedicels 0.6–1.0(–1.2) mm long, ± glabrous (sometimes bearing sparse, minute, antrorse prickle-teeth), ± appressed to branchlets. Spikelets 2.8–3.0 mm, 1(–2)-flowered, lanceolate, light green to stramineous. Glumes green (± hyaline), glabrous, ovate-lanceolate to ovate, acute to subacute, 1-nerved, lower and upper glume margins entire, ciliate; lower glume 0.3–1.0 mm, upper glume 1.0(–1.6) mm. Lemma 2.6–3.0 mm, light green to grey, scabrid (densely covered in minute prickle-teeth), 3(–5)-nerved, ovate-lanceolate, acute to mucronate, sometimes with a minute subapical awnlet 0.06-0.08 mm long. Palea 2.4–2.8 mm, scabrid (densely covered in minute prickle-teeth), lanceolate, pale green to green, pubescent, 1–2-nerved. Rachilla prolongation 0.3–0.5 mm, filiform, hyaline, glabrous. Stamens 3. Filaments 0.3 mm long, hyaline. Anthers 0.7–1.5 mm, purple or yellow. Ovary narrowly ovoid to weakly trigonous 1.0–1.25 mm long, green, ± glabrous (basal portion sometimes minutely ciliate); styles apical, 0.10–0.25 mm, hyaline; stigmas plumose, white. Caryopsis 1.3–1.5 mm long, laterally compressed, orange when mature. Chromosome number: 2*n* = 28 (Zotov 1971).

##### Specimens seen.

North-West Nelson, Gouland Downs, *A.P. Druce s.n.*, Jan 1969, WELT SP069213; North-West Nelson, Cobb Valley, Chaffey’s Stream, *A.M. Hamilton s.n.*, 23 Feb 1965, CHR 119546; North-West Nelson, Peel Range, above Lake Henderson, *A.P. Druce s.n.*, Mar 1982, CHR 369973; North-West Nelson, below Balloon Hut, *I.M. Ritchie s.n.*, 29 Mar 1967, CHR 175738; North-West Nelson, Kahurangi National Park, Cundy Creek, *M.J. Thorsen 101/09*, 11 Apr 2009, AK 304802; North-West Nelson, Kahurangi National Park, west of Gordon’s Pyramid, *M.J. Thorsen 106/09*, 12 Apr 2009, AK 305801; North-West Nelson, Mt Arthur, ?*A. Mackay s.n.*, c.1879, CHR 13277; North-West Nelson, South Arthur Range, west side of Baton Saddle, *A.P. Druce 478*, Feb 1991, CHR 469403; North-West Nelson, Lockett Range, near Ruby Lake, *A.P. Druce s.n.*, Jan 1982, CHR 387666–387667; North West of Mt FZ (between Glenroy and Sheriff Rivers), *A.P. Druce s.n.*, 13 Mar 1984, CHR 394262.

##### Distribution

**(Fig. 1).** *Simplicia buchananii* is endemic to North-West Nelson, South Island.

##### Recognition.

*Simplicia buchananii* is distinguished from *Simplicia laxa* and *Simplicia felix* by the tufted growth habit, erect culms, and by the linear inflorescences, whose branches are usually tightly appressed to the rachises (Fig. 5, for other differences see Table 1).

##### Ecology.

*Simplicia buchananii* is a biologically sparse species of calcareous rock habitats (including shaded rock outcrops, boulderfalls, rock overhangs and cave entrances) within montane forest (de Lange et al. 2010).

##### Conservation status.

Using the New Zealand Threat Classification System (Townsend et al. 2008) *Simplicia buchananii* has been assessed as ‘Threatened / Nationally Critical’ with the qualifiers ‘DP’ [Data Poor], ‘RR’ [Range Restricted] and ‘Sp’ [Sparse] (de Lange et al. 2013). The threats this species faces were summarised by de Lange et al. (2010) and Smissen et al. (2011). Based on our current knowledge of this species the current threat status remains appropriate.

#### Simplicia laxa

Taxon classificationPlantaePoalesPoaceae

Kirk, T.N.Z.I. 29: 497, t.44 (1897)

≡ Simplicia laxa Kirk var. laxa (autonym, Zotov T.R.S.N.Z. 73: 236 (1943))

##### Lectotype.

‘Waikouaiti, Otago’ *D. Petrie s.n.*, n.d. (WELT SP043017!) (*fide* Zotov 1971)

##### Isolectotype.

‘Waikouaiti, Otago, *D.Petrie s.n.*, n.d. (WELT SP043021!) (*fide* Zotov 1971)

##### Etymology.

Kirk (1897) did not explain the meaning of his species epithet ‘*laxa*’ though his intent is clear from his protologue where he describes the new species as having ‘weak, decumbent, flaccid’ culms. The epithet is derived from Latin ‘*laxus*’ meaning ‘loosely arranged’ as in ‘wide, loose’ structures or growth (Taylor 2002).

##### Description

**Figure 6.**
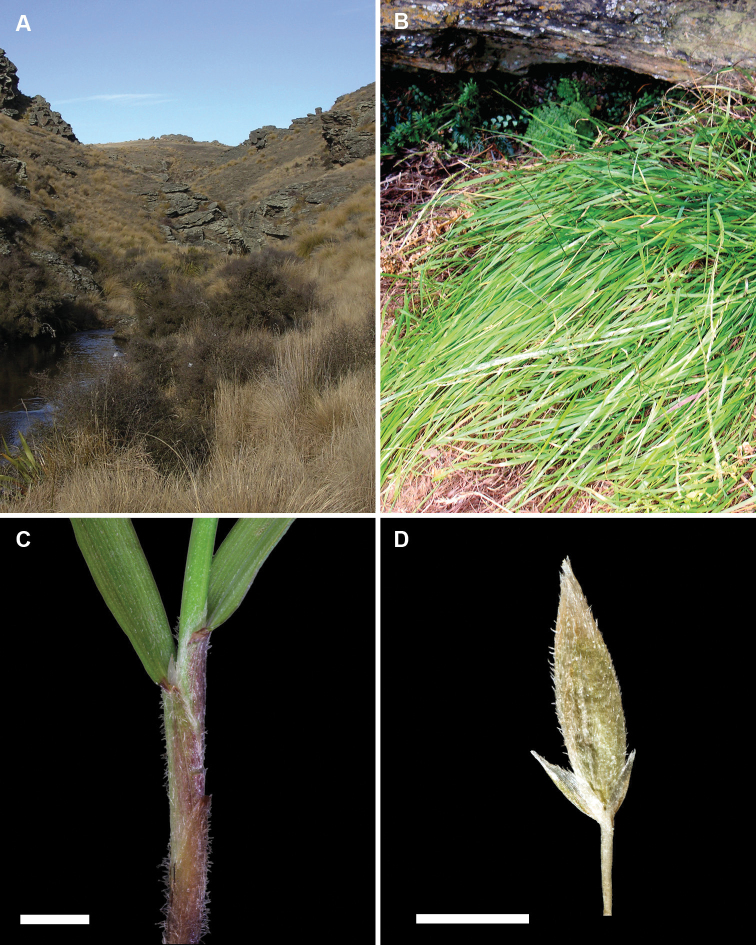
*Simplicia laxa*. **A** habitat, Emerald Stream, McCraes, North Otago, South Island, New Zealand (image D.A. Houston) **B** growth habit and inflorescence **C** culm, leaf base, sheath and ligule **D** spikelet showing reduced glumes and lemma. (Photo credit images **C** and **D**: K. Ford, Allan Herbarium, Landcare Research Manaaki Whenua)

**(Fig. 6).** Plants trailing forming thick sprawling mats or diffuse interconnected patches up to 0.6 m across. Culms 0.40–0.80 m long, green to pale-green when fresh, wiry, decumbent, with the apices weakly erect, culm internodes 4–8, elongated, sparsely (sometimes densely) hairy, or glabrous; hairs weakly flexuous, patent up 0.18 mm long; internodes usually shorter than subtending leaf-sheaths. Culm-nodes conspicuously swollen when fresh, maroon-black to black (0.13–)0.18–0.30 mm long, rooting freely on contact with ground. Basal leaf-sheaths glossy light brown to amber, membranous, ribbed, abaxially (often copiously) pubescent on ribs (and usually on interstices), hairs 0.20–0.25(–0.30) mm long, patent to retrorse; mid stem and upper leaf-sheaths pale-green to green, membranous, ribbed, abaxially pubescent on ribs (and sometimes on interstices), hairs copious, 0.35–0.40 mm long patent, mostly straight, sometimes curved or weakly flexuous. Ligule 2.8–3.5(–10) mm, membranous, lanceolate, apex erose to very deeply lacerate; abaxially sparsely to copiously hairy; hairs 0.20–0.24 mm long. Leaf-blade (100–)160(–200) × (2.8–)3.0(–3.6) mm, green to dark green, flat, linear-lanceolate, finely ribbed; adaxial ribs finely pubescent, abaxially glabrous (sometimes sparsely hairy at leaf base; margins ± smooth, sometimes irregularly finely scabrid and sparsely hairy. Panicle (40–)100(–150) mm long, linear to ± pyramidal, usually with basal branch or branch pair reflexed (often unevenly so); rachis glabrous, branches (20–)40(–60) mm long, finely, antrorsely hairy (hairs 0.20–0.25 mm long), binate, initially contracted but as inflorescences mature, spreading to reflexed, devoid of spikelets in lower half; pedicels appressed to branchlets, 1.00–1.06 mm long, finely pubescent. Spikelets 2.8–3.2 mm, 1-flowered, lanceolate, light green. Glumes pale green (± hyaline), glabrous, broadly ovate-lanceolate to ovate, acute, 1-nerved, nerve extending beyond apex as a minute mucro, lower glume margins entire (sometimes with apex erose), ciliate towards apex, upper glume margins usually erose (sometimes subentire), ciliate; lower glume 0.5–0.8 mm, upper glume 0.75–1.0(–1.2) mm. Lemma 2.8–3.2(–3.4) mm, light green to grey-green (sometimes purple-green), ± evenly, densely pubescent, lanceolate, acute, apex mucronate (mucro 0.10–0.25 mm long), 3(–5)-nerved (nerves obscured by hairs); lemma hairs antrorse appressed, sericeous, 0.12–0.13 mm long. Palea 2.4–2.8 mm, lanceolate, pale green to green, pubescent, 1–2-nerved, (nerves obscured by hairs). Rachilla prolongation 1.25–1.30 mm, narrowly lanceolate, hyaline, margins minutely ciliate. Stamens 3. Filaments 0.20–0.25 mm long, hyaline. Anthers 0.30–0.45 mm, yellow. Ovary narrowly ovoid to weakly trigonous 1.0–1.25 mm long, dark green, ± glabrous (basal portion sometimes minutely ciliate); styles apical, 1.10–1.25 mm, hyaline; stigmas plumose, white. Caryopsis 1.4–1.5 mm long, laterally compressed, orange-brown when mature. Chromosome number: 2*n* = 28 (Zotov 1971, *I.M. Ritchie s.n.*, CHR 202752)

##### Specimens seen.

Karamea, Honeycomb Cave, *P. Wardle s.n.*, 22 Jan 1985, CHR 489550; Karamea Ecological District, Karamea, Kahurangi National Park, Honeycomb Cave, *P.J. de Lange 4774*, 6 Dec 2000, AK 252968; Otago, Taieri County, near Deep Stream Hotel, Rock & Pillar Road, *D. Petrie s.n.*, Feb 1877, WELT SP010498, WELT SP043018, WELT SP043020A, WELT SP043020B, WELT SP043020C, WELT SP069211; Old Man Ecological District, Old Man Range, Castle Rock Summit, *P.J. de Lange 7859*, 18 Jan 2008, AK 304848 (Duplicate: US); Old Man Ecological District, Old Man Range, Castle Rock Summit (south side), *P.J. de Lange 7858*, 18 Jan 2008, AK 304847; Macraes Ecological District, Nenthorn, Deighton Stream tributary, ‘John’s Cave’, *P.J. de Lange 7206 & M.J. Thorsen*, 15 Jan 2008, AK 301580; Macraes Ecological District, Nenthorn, Upper Emerald Stream, *P.J. de Lange 7835 & M.J. Thorsen*, 15 Jan 2008, AK 304808; Macraes Ecological District, Nenthorn, Upper Emerald Stream, *P.J. de Lange 7836 & M.J. Thorsen*, 15 Jan 2008, AK 304809; Macraes Ecological District, Nenthorn, Emerald Stream, ‘Old Otagense site’, *P.J. de Lange 7856 & M.J. Thorsen*, 15 Jan 2008, AK 304845 (Duplicate: US); Summerhills Station, 3 O’Clock Stream, *M.J. Thorsen 100/07*, 14 May 2007, CHR 591912; Nevis Valley, Barn Creek, *G. Loh s.n.*, 26 Nov 1994, CHR 509148. CULTIVATED: Ex. Cult., Karamea Ecological District, Karamea, Kahurangi National Park, Honeycomb Cave, *P.J. de Lange 6104*, 3 Sep 2004, AK 288071 (Duplicate: HO). Macraes Ecological District, north-east of Nenthorn, Macraes, Emerald Stream, T Whitaker Falcon Site, *M.J. Thorsen s.n.*, 30 Mar 2006, AK 295631; Ex. Cult., Central Otago, Old Man Range, Castle Rock, *I.M. Ritchie s.n.*, 25 Oct 1969 (grown on and harvested by V.D. Zotov on 31 Dec 1970 as G8128).

##### Distribution

**(Fig. 1).** As recircumscribed here *Simplicia laxa* is now endemic to the South Island. Nevertheless, there are a few historical collections held in world herbaria that suggest that *Simplicia felix* and *Simplicia laxa* once grew sympatrically in the eastern Wairarapa. de Lange (2016) has shown that these herbarium specimens are the result of accidental mixing of unmounted Thomas Kirk, North Island (*Simplicia felix*) and Donald Petrie, South Island (*Simplicia laxa*) specimens, and mislabelling by Thomas Cheeseman and, possibly, Victor Zotov, rather than genuine North Island wild occurrences of *Simplicia laxa*. *Simplicia laxa* is currently known from one site near Karamea, North-West Nelson (Honeycomb Cave) and otherwise from 10 sites in Northern and Central Otago (Smissen et al. 2011). This disjunct distribution is unlikely to be natural, however, it more likely reflects the loss of interconnecting habitat as well as the difficulty of recognising this species in the field.

##### Recognition.

Smissen et al. (2011) showed that *Simplicia laxa* is more closely related to *Simplicia felix* than it is to *Simplicia buchananii*. From *Simplicia felix*, *Simplicia laxa* can be distinguished by the culm internodes which are shorter than the subtending leaf sheaths and usually hairy (sometimes glabrous); and by the less strongly ribbed, glossy light brown to amber basal sheaths, whose ribs are pubescent (hairs 0.20–0.30 mm long). The mid-stem and upper-stem leaf sheaths of *Simplicia laxa* are less prominently ribbed than those of *Simplicia felix* and the ribs and, usually the interstices are hairy. The adaxial leaf surface ribs of *Simplicia laxa* are hairy (abaxially glabrous), while the leaf margin is mostly smooth, though sometimes irregularly hairy or finely scabrid. The leaves of *Simplicia laxa* are also wider than those of *Simplicia felix* (2.8–3.6 mm wide cf. 1.0–3.0 mm wide in *Simplicia felix*). However, in cultivation the leaves of both species can get up to 4.0 mm wide. Although the inflorescences of *Simplicia laxa* and *Simplicia felix* are similar, those of *Simplicia laxa* are larger (up to 150 mm rather than 80 mm long), and the branches are antrorsely hairy rather than scabrid. Although the lemma of both species overlap in range, those of *Simplicia laxa* are longer (2.8–3.4 mm long) than those of *Simplicia felix* (2.0–3.0 mm long) and minutely pubescent rather than scabrid. The rachilla prolongation of *Simplicia laxa* is narrowly lanceolate, 1.25–1.30 mm long and with the margins minutely ciliate, while that of *Simplicia felix* is filiform, 0.8 mm long; bearing sparse cilia only near the apex. Other differences are given in Table 2.

**Table 2.** Distinguishing features of *Simplicia* species based on wild collected material.

	*Simplicia buchananii*	*Simplicia laxa*	*Simplicia felix*
Growth habit	Tufted, erect. culms up to 1 m tall	Decumbent, culms sprawling, forming mats up to 0.6 m diameter	Decumbent, sprawling, forming mats up to 1 m diameter
Culm internodes	Glabrous, ± equal in length to subtending leaf sheaths	Hairy or glabrous, < subtending leaf-sheaths. Hairs if present up to 0.18 mm long	Glabrous, > subtending leaf-sheaths
Culm nodes	Glossy orange-brown to dark red-brown	Glossy maroon-black to black	Glossy dark brown-green to brown-black
Basal leaf sheaths	Strongly ribbed, stramineous or dull brown, glabrous or with ribs scabrid (very rarely finely pubescent). Hairs (if present) 0.06–0.08 mm long	Finely (‘weakly’) ribbed, glossy light brown to amber, ribs pubescent. Hairs 0.20–0.30 mm long	Strongly ribbed, dull dark brown, ribs glabrous or pubescent. Hairs (if present) 0.10–0.15 mm long
Mid-stem and upper-stem leaf sheaths	Strongly ribbed. Glabrous	Hairy. Hairs copious, 0.35–0.40 mm long	Strongly ribbed. Usually glabrous, occasionally ribs finely short pubescent towards sheath apex
Ligule	Glabrous	Sparsely to copiously hairy. Hairs 0.20–0.24 mm long	Glabrous or with both surfaces finely hairy. Hairs 0.15–0.18 mm long
Leaf-blade	1.8–4.0 mm wide. Adaxially finely scabrid, abaxially glabrous, margins smooth. Apex acuminate	2.8–3.6 mm wide. Adaxially with hairy ribs, abaxially glabrous; margins ± smooth, sometimes, finely scabrid and/or sparsely hairy. Apex acute	1.0–3.0 mm wide. Ribs (both surfaces) smooth or finely scabrid; margins smooth or finely scabrid. Apex acute
Inflorescence	Paniculate, linear up to 180 mm long. Branches glabrous, erect, appressed to rachis, bearing spikelets almost to base.	Paniculate, linear to ± pyramidal, up to 150 mm long. Branches antrorsely hairy, basal branches (or branch) reflexed, others weakly appressed to rachis, lower half of branch devoid of spikelets	Paniculate, linear to ± pyramidal, up to 80 mm long. Branches scabrid, basal branches (or branch) reflexed, others weakly appressed to rachis, lower ½ to ⅔ or branch devoid of spikelets
Pedicels	Glabrous, 0.60–1.2 mm long	Pubescent, 1.00–1.06 mm long	Pubescent 0.20–0.30 mm long
Glumes	Lower glume 0.3–1.0 mm long, upper glume 1.0–1.6 mm long	Lower glume 0.5–0.8 mm long, upper glume 0.75–1.2 mm long	Lower glume 0.5–0.6 mm long, upper glume 0.75–0.9 mm long
Lemma	2.6–3.0 mm long, scabrid	2.8–3.4 mm long, pubescent	2.0–3.0 mm long, minutely scabrid
Rhacilla prolongation	0.3–0.5 mm long, filiform, glabrous	1.25–1.30 mm long, narrowly lanceolate, margins minutely ciliate	0.8 mm long, filiform, glabrous except for sparse cilia cresting apex
Anther filaments	0.3 mm long,	0.20–0.25 mm long	0.6–0.9 mm long
Anthers	0.7–1.5 mm long,	0.30–0.45 mm long	1.0–1.2 mm long

Because of the lax, decumbent trailing growth habit, and loose linear to pyramidal inflorescences *Simplicia laxa* is easily distinguished in the field from the shortly tufted *Simplicia buchananii* whose inflorescences are erect and whose inflorescence branches are held tightly appressed to the rhacis (Zotov 1971; Edgar and Connor 2010; de Lange et al. 2010). Other differences are provided here in the key to the species and under Table 2.

##### Ecology.

Much of what has been written about *Simplicia laxa* (Johnson 1995; de Lange et al. 2010) we now believe is based on observations made of remnant populations persisting in possibly suboptimal habitats (rock overhangs, rock crevices, river gorges) within locations that had once been forested. Nevertheless, the North-West Nelson, Honeycomb Cave population, which occurs in dense lowland forest, is still confined to a cave entrance and the species has yet to be found within the surrounding forest. It seems likely that *Simplicia laxa* is a species of deeply shaded habitats, which may, like *Simplicia felix* also occur in forested situations. Further survey is needed. In the interim, all of the extant *Simplicia laxa* populations occur on base-rich substrates, chlorite schist and limestone, and in having this in common with the other two species it is unlikely that it will be found on less fertile substrates.

##### Conservation status.

*Simplicia laxa* was assessed as ‘Threatened / Nationally Critical’ qualified ‘CD’ (Conservation Dependent), ‘Sp’ (Sparse) by de Lange et al. (2013). That assessment included plants described here as *Simplicia felix*. With the recircumscription of *Simplicia laxa* the species remains appropriate assessed as ‘Nationally Critical’. Currently there are < 15 populations known, and several of these are in decline, and very few are substantial in size. Many occur on private land without direct conservation management or in places subject to ongoing habitat deterioration through invasive weed pressure and habitat loss. The qualifiers however need adjustment. Because the species is managed in a number of sites it is appropriate to retain the qualifier ‘CD’, as ceasing management would have a serious impact on the survival of the species. It is debatable whether *Simplicia laxa* is truly biologically sparse. It is sparsely distributed but this is more likely an artefact of past habitat loss leaving highly fragmented, disjunct ‘remnant’ populations rather than any natural pattern of distribution or species biology. We recommend that ‘Sp’ be removed from the conservation assessment for this species. The seemingly peculiar North-West Nelson, Honeycomb Cave outlier suggests that *Simplicia laxa* should be looked for throughout the South Island rather than, as it currently has, only in the Central and Eastern Otago Region. Also, it is now evident that we lack trend data for the species, though the overall impression is that many populations are in decline. For these reasons, we recommend that the species be qualified ‘DP’ (Data Poor) be added to the species conservation status.

#### Simplicia felix

Taxon classificationPlantaePoalesPoaceae

de Lange, J.R.Rolfe, Smissen & Ogle sp. nov.

urn:lsid:ipni.org:names:77159000-1

##### Diagnosis.

Differs from *Simplicia laxa* by dark brown, prominently ribbed leaf sheaths; mostly glabrous, strongly ribbed mid-stem to upper-stem leaf sheaths; longer culm internodes; narrower, glabrous (sometimes with the adaxial ribs finely scabrid) leaves; shorter panicles (up to 80 mm long) with scabrid branches; minutely scabrid lemma and smaller filiform rachilla prolongation bearing cilia only at the apex.

##### Holotype

**Figure 7.**
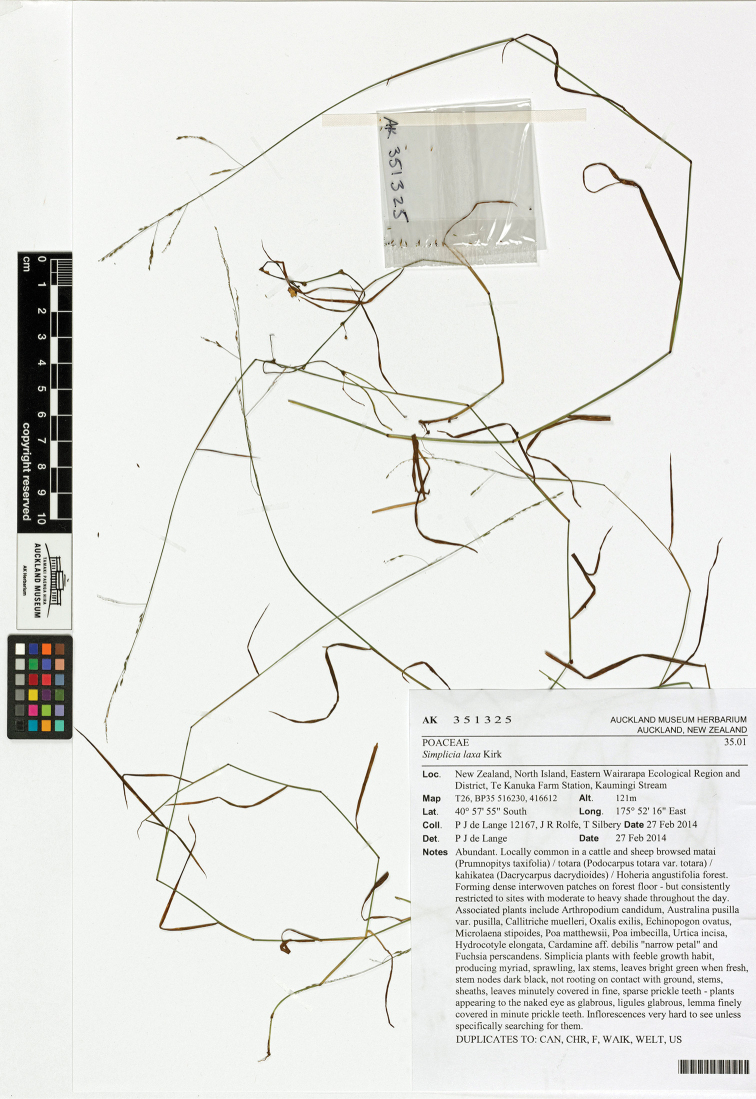
Holotype of *Simplicia felix* de Lange, J.R.Rolfe, Smissen & Ogle.

**(Fig. 7).** ‘Eastern Wairarapa Ecological Region and District, Te Kanuka Farm Station, Kaumingi Stream’ *P.J. de Lange 12167, J.R. Rolfe & T. Silbery*, 27 Feb 2014, (AK 351325) Isotypes. CAN, CHR, F, WAIK, WELT, US

##### Etymology.

The epithet ‘*felix*’ is taken from the Latin for ‘*lucky*’ (N.G. Walsh, MEL pers comm., 14 January 2016) as in ‘*lucky find*’ in reference to the circumstances of this species’ discovery; that came about through the desire to get a name on an unremarkable little tuft of grass that was discovered fortuitously near Mangaweka, Central North Island by CCO on 29 January 2005 (Ogle 2010).

##### Description

**Figure 8.**
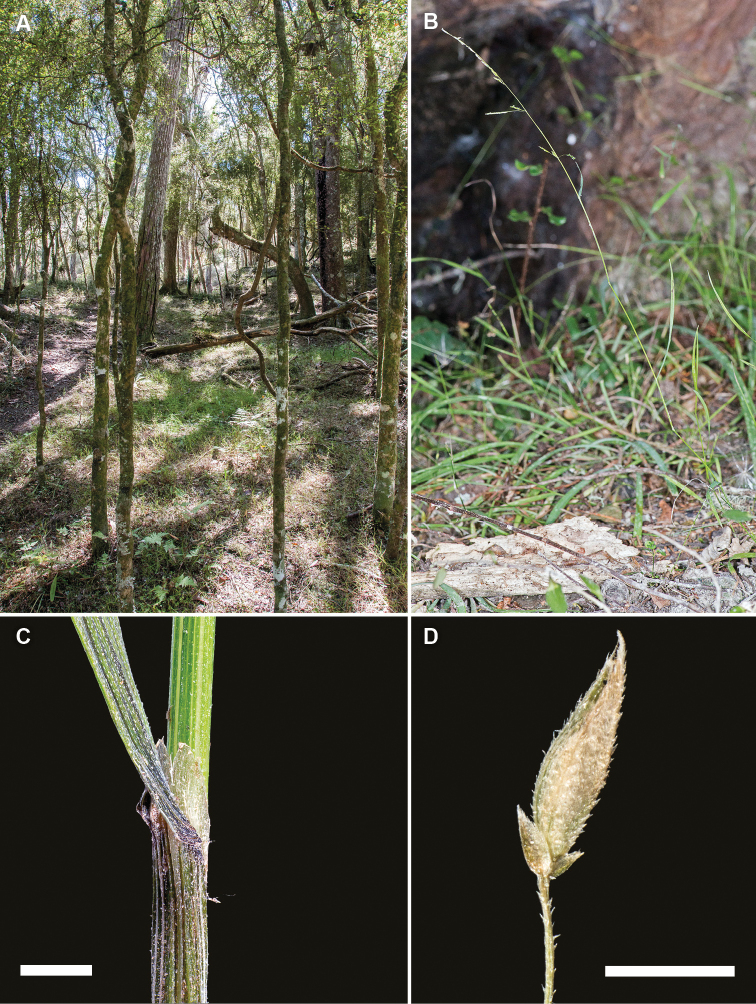
*Simplicia felix*. **A** habitat, Te Kanuka Farm Station, Upper Kaumingi Stream east Wairarapa, North Island, New Zealand **B** growth habit and inflorescence **C** culm, leaf base, sheath and ligule **D** spikelet showing reduced glumes and lemma (images: J.R. Rolfe)

**(Fig. 8).** Plants forming flaccid, diffuse, often much interconnected, sprawling patches up to 1 m across. Culms 0.25–0.65 m long, green to dark brown when fresh, wiry, initially decumbent, becoming ascendant with the apices weakly erect, culm internodes 5–8, elongated, glabrous; internodes longer than subtending leaf-sheaths. Culm-nodes conspicuously swollen when fresh, dark green-brown to brown-black 0.15–0.25 mm long, rooting freely on contact with ground. Basal leaf-sheaths dull dark brown, membranous, strongly ribbed, usually abaxially pubescent (sometimes glabrous) on ribs, hairs 0.10–0.15 mm long, patent to retrorse; mid stem and upper leaf-sheaths pale-green to green, membranous, strongly ribbed, glabrous (rarely abaxially ribs finely pubescent toward sheath apex). Ligule 2.0–2.6 mm, membranous, lanceolate, apex entire, or deeply lacerate; glabrous, or with both surfaces hairy; hairs 0.15–0.18 mm long. Leaf-blade (20–)40(–60) × (1.0–)1.2–2.4(–3.0) mm, yellow-green to dark green, flat, narrow linear-lanceolate, finely ribbed, ribs smooth (sometimes minutely scabrid); margins minutely scabrid. Panicle 20–40(–80) mm long, linear to ± pyramidal, usually with basal branch or branch pair reflexed (often unevenly so); rachis glabrous (sometimes bearing a few minute prickle-teeth), branches 20–30 mm long, scabrid, binate, initially contracted but as inflorescences mature, spreading to reflexed, devoid of spikelets in lower half to two-thirds; pedicels appressed to branchlets, 0.20–0.25(0.30) mm long, finely pubescent. Spikelets 2.7–3.0 mm, 1-flowered, lanceolate, light green. Glumes pale green (± hyaline), glabrous, ovate-lanceolate to ovate, acute, 1-nerved, nerve sometimes extending beyond apex as a minute mucro, margins initially entire, becoming erose near apex, very sparsely ciliate in upper third; lower glume 0.5–0.6 mm, upper glume 0.75–0.8(–0.9) mm. Lemma 2.0–2.8 (–3.0) mm, light green to cream, ovate-lanceolate to lanceolate, acute, apex mucronate (mucro 0.1 mm long), 5-nerved, the inner 3 nerves conspicuous, the outer less prominent; nerves bearing evenly spaced minute (0.02–0.03 mm long), antrorse, appressed prickle-teeth, interstices usually densely (sometimes sparsely) covered with minute antrorse prickle-teeth. Palea 2.0–2.8 mm, lanceolate, green to purple-green, 1–2-nerved, nerves bearing evenly spaced minute prickle (0.02–0.03 mm long) teeth, interstices usually glabrous, sometimes sparsely covered with minute prickle-teeth. Rachilla prolongation 0.8 mm, filiform, hyaline, glabrous except for sparse cilia cresting prolongation apex. Stamens 3. Filaments 0.6–0.9 mm long, hyaline. Anthers 1.0–1.2 mm, yellow. Ovary narrowly ovoid to weakly trigonous 1.0 mm long, pale green, glabrous; styles apical, 1.0–1.2 mm, hyaline; stigmas plumose, white. Caryopsis 1.2–1.4(–1.5) mm long, laterally compressed, pale orange to orange-brown when mature. Chromosome number: 2*n* = 28 (Murray et al. 2005, *P.J. de Lange 5897*, AK 285424—as *Simplicia laxa*)

##### Specimens seen.

New Zealand, North Island: Rangitikei Ecological Region and District, North of Taihape, north of Paengaroa Road and east of State Highway One, Ngawaka Stream, ‘Stevies Bush’, *P.J. de Lange 7834 & C.C.Ogle*, 29 Feb 2008, AK 304807 (Duplicates: CHR, US); Rangitikei Ecological Region and District, North of Taihape, south of Paengaroa Road and east of State Highway One, *C.C. Ogle 4955*, 17 Feb 2006, AK 295628; Rangitikei Ecological Region and District, North of Taihape, south of Paengaroa Road and east of State Highway One, *C.C. Ogle 4954*, 17 Feb 2006, AK 295627; Rangitikei Ecological Region and District, North of Taihape, south of Paengaroa Road and east of State Highway One, ‘Campbells Bush’ *P.J. de Lange 7832* & *C.C. Ogle*, 29 Feb 2008, AK 304805; Taihape, Oraukura Stream, *C.C. Ogle 5625*, 6 Apr 2008, AK 306012; Rangitikei Ecological Region and District, Taihape Scenic Reserve, Hautapu, *P.J. de Lange 7833*, *C.C. Ogle & V. McGlynn*, 28 Feb 2008, AK 304806; Rangitikei Ecological Region and District, Taihape Scenic Reserve, Hautapu, *C.C. Ogle 4958 & V. McGlynn*, 7 Mar 2006, AK 297357; Rangitikei Ecological Region and District, Rangitikei, Kawhatau Valley, Toetoe Road, *C.C. Ogle 4893, V. McGlynn & G. La Cock*, 13 Dec 2005, AK 297353; Rangitikei Ecological Region and District, Rangitikei, Kawhatau Valley, Toetoe Road, *P.J. de Lange 7831*, *C.C. Ogle & V. McGlynn*, 28 Feb 2008, AK 304804; Rangitikei Ecological Region and District, Rangitikei, Kawhatau River, Toetoe Road, ‘Ben Moi’ farm, *C.C. Ogle 4734*, 29 Jan 2005, AK 289755 (Duplicate: CHR); Eastern Wairarapa Ecological Region and District, Te Kanuka Farm Station, Swamp Ridge Covenant, *P.J. de Lange 12165, J.R. Rolfe & T. Silbery*, 27 Feb 2014, AK 351320 (Duplicates lodged in: CHR.WELT, US); Eastern Wairarapa Ecological Region and District, Te Kanuka Farm Station, Upper Kaumingi Stream, *P.J. de Lange 12168, J.R. Rolfe & T. Silbery*, 27 Feb 2014, AK 351330 (Duplicates lodged in: CAN,WELT, US); Wairarapa, Ruamahanga, *T. Kirk s.n.*, *n.d*., WELT SP043016; Ruamahanga Valley, *T. Kirk s.n.*, 26 Jan 1880, WELT SP043022; Eastern Wairarapa Ecological Region and District, Admiral Road, Wainuoru River, Te Kowhai, Moetapu Bush, *P.J. de Lange 12160, J.R. Rolfe & T. Silbery*, 26 Feb 2014, AK 351290 (Duplicates: WELT, US); Eastern Wairarapa, Longbush, Tawhiriwaimanuka Stream. Ahipaku QE II Covenant, *J. R. Rolfe 15017*, 9 Dec 2015, AK 360429 (Duplicate: WELT). South Island: South-East of Duntroon, Prydes Gully Road (Ngapara, The Knolls), *B.P.J. Molloy s.n.*, 18 Dec 1991, CHR 616708; Duntroon Ecological District, Ngapara, The Knolls, *P.J. de Lange 1340 & B.P.J. Molloy*, 7 May 1992, AK 208577 (Duplicates: CHR, WAIK, WELT). Cultivated: Ex. Cult., Rangitikei Ecological Region and District, Rangitikei, Kawhatau Valley, Toetoe Road, P.J. de Lange 6791, 30 Nov 2006, AK 297927; Ex. Cult., Rangitikei Ecological Region and District, Rangitikei, Kawhatau Valley, Toetoe Road, P.J. de Lange 6824, 23 Dec 2006, AK 298065; Ex. Cult., Duntroon Ecological District, Ngapara, The Knolls, *P.J. de Lange 5897*, 23 Feb 2004, AK 285424; Ex. Cult., Duntroon Ecological District, Ngapara, The Knolls, *P.J. de Lange 6103*, 3 Sep 2004, AK 288070.

##### Distribution

**(Fig. 1).** *Simplicia felix* has so far been collected from the North and South Islands, from the current northern limit at Ngawaka Stream, near Taihape, North Island to Ngapara, North Otago, South Island. In the North Island the species has been found in two broad geographic areas, around Taihape–Mangaweka, and in the eastern Wairarapa. In the South Island, *Simplicia felix* is so far known only from the one location at Ngapara.

##### Recognition.

Genetically and morphologically *Simplicia felix* is more closely related to *Simplicia laxa* than it is to *Simplicia buchananii*. From *Simplicia laxa* it can be distinguished by the culm internodes which are longer than the subtending leaf sheaths and consistently glabrous; and by the strongly ribbed, dull dark brown basal sheaths, whose ribs are glabrous or pubescent (if pubescent then with the hairs 0.10–0.15 mm long). The mid-stem and upper-stem leaf sheaths of *Simplicia felix* are strongly ribbed and usually glabrous (occasionally the ribs are finely pubescent towards the sheath apex). The leaf surfaces and margins of *Simplicia felix* are mostly smooth though the ribs and leaf margins may be minutely scabrid. As a rule, the leaves of *Simplicia felix* are also shorter (up to 60 mm in *Simplicia felix*, 200 mm in *Simplicia laxa*) and narrower than those of *Simplicia laxa* (1.0–3.0 mm long cf. 2.8–3.6 mm long in *Simplicia laxa*). However, in cultivation the leaves of both species can get up to 4.0 mm wide. Although the inflorescences of *Simplicia laxa* and *Simplicia felix* are similar, those of *Simplicia felix* are smaller (up to 80 mm long rather than 150 mm long), and the branches are scabrid rather than antrorsely hairy. Although the lemma of both species overlap in range, those of *Simplicia felix* tend to be shorter (2.0–3.0 mm long) than those of *Simplicia laxa* (2.8–3.4 mm long) and minutely scabrid rather than pubescent. The rachilla prolongation of *Simplicia laxa* is narrowly lanceolate, 1.25–1.30 mm long and with the margins minutely ciliate, while that of *Simplicia felix* is filiform, 0.8 mm long and bearing sparse cilia only near the apex. Other differences are given in Table 2.

*Simplicia felix* was initially confused with *Simplicia buchananii* (see Ogle 2010; Smissen et al. 2011) mostly because the culm internodes of both species are glabrous; they have superficially similar strongly-ribbed, often glabrous leaf-sheaths, similar smooth or finely scabrid leaves, and minutely scabrid lemma. Despite these similarities, *Simplicia felix* is not closely allied to *Simplicia buchananii* from which it is readily distinguished by the lax, sprawling, rather than erect culms; linear-pyramidal rather than linear inflorescences with binate branching, and by the lower branch or branches usually reflexed rather than appressed to the rachis. The pedicels of *Simplicia felix* are also pubescent rather than glabrous (rarely minutely scabrid).

##### Ecology.

With the exception of the Ngapara population which grows within a limestone overhang, *Simplicia felix* has only been collected from central and eastern North Island lowland to lower montane, often riparian, seasonally dry (drought prone), Podocarp forests overlying base-rich substrates such as limestone, calcareous mudstone and siltstone. In these areas plants have been found only in lightly shaded situations within forest remnants that have been lightly under-grazed by cattle or sheep (Ogle 2010; de Lange et al. 2014). The reduced competition from other grasses and herbs that grazing causes, seems crucial to this species’ survival (de Lange et al. 2014) as, in some locations where *Simplicia felix* had been found, portions of the same forest that had been fenced to exclude livestock did not have *Simplicia*). In the Taihape–Rangitikei area, Ogle (2010) noted a close association with a range of other indigenous grasses and herbs, most notably *Echinopogon ovatus*, *Poa imbecilla*, *Poa matthewsii*, *Cardamine debilis* agg., *Oxalis exilis*. In the Eastern Wairarapa the same species along with *Arthropodium candidum*, Australina pusilla subsp. pusilla, *Stellaria parviflora* and the mosses *Camptochaete angustata*, *Echinodium hispidum* and *Plagiomnium novae-zelandiae* were key associates of *Simplicia felix* (P. J. de Lange unpubl. data). Further survey of similar forest remnants with ground covers dominated by these species is, we believe, likely to locate further populations.

The Ngapara site, as *Simplicia laxa*, was described in some detail by Johnson (1995). By comparison with the North Island sites it is anomalous and it seems likely that *Simplicia* persists there because the rock overhang affords some shade and protection from competing plants. Within the rock overhang *Simplicia felix* grows with *Poa imbecilla*, *Poa matthewsii*, *Chenopodium allanii* and an unnamed member of the *Cardamine corymbosa* complex.

##### Conservation status.

*Simplicia felix* occupies a very small area of only a few square metres wherever it occurs. It appears to have quite specific light requirements and tolerates only limited competition from other ground-cover species. The healthiest populations occur at sites where competition is reduced by grazing mammals such as cattle and sheep. This poses a quandary for conservation managers because, whilst grazing apparently benefits *Simplicia felix*, it will ultimately lead to the collapse of the forest canopy that provides the level of shade that is also necessary for *Simplicia felix* to survive. Collectively, the area of the sites where *Simplicia felix* occurs amounts to considerably less than 1 ha. Therefore, *Simplicia felix* meets the criteria to be assessed Threatened—Nationally Critical B2 using the New Zealand Threat Classification System (Townsend et al. 2008). We also recommend that the qualifiers Data Poor (‘DP’) and Range Restricted (‘RR’) be appended to the assessment—‘DP’ because there are no population trend data available and because several areas of possibly suitable habitat in the Rangitikei and Eastern Wairarapa have not been surveyed for the presence of *Simplicia felix*; ‘RR’ because its habitat requirements are apparently very narrow.

## Supplementary Material

XML Treatment for Simplicia

XML Treatment for Simplicia buchananii

XML Treatment for Simplicia laxa

XML Treatment for Simplicia felix
